# “Effects of Tobacco Smoke on Aeroallergen Sensitization and Clinical Severity among University Students and Staff with Allergic Rhinitis”

**DOI:** 10.1155/2020/1692930

**Published:** 2020-10-08

**Authors:** Theerapan Songnuy, Stephen J. Scholand, Sarawut Panprayoon

**Affiliations:** ^1^Department of Clinical Medical Science, Walailak University School of Medicine, Nakhon Si Thammarat 80160, Thailand; ^2^Department of Medicine, University of Arizona, Tucson, AZ 85724, USA; ^3^Department of Social Medicine, Walailak University Hospital, Nakhon Si Thammarat 80160, Thailand

## Abstract

Allergic diseases, affecting a variety of organs, have continuously increased both in developed and developing countries. Tobacco smoke exposure increases prevalence of allergic rhinitis (AR) and may affect allergic sensitization. This study was designed to compare indoor-aeroallergen sensitization between those not exposed and exposed to tobacco smoke in university students and staff with allergic rhinitis. A cross-sectional descriptive study among university students and staff with allergic rhinitis was performed from February 1, 2018, to March 31, 2019. Questionnaires regarding demography, clinical symptoms, and tobacco smoke exposure were implemented. A current smoker was defined as using, at least, 1 cigarette per day for, at least, 1 month. A secondhand smoker was defined as the one who never smoked, but lived with a current smoker, at least, for 1 month. A skin prick test for eight common indoor aeroallergens, *Dermatophagoides pteronyssinus*, *Dermatophagoides farinae*, *Periplaneta americana*, cat dander, dog dander, para grass, careless weed, and *Cladosporium* spp., was performed. Sensitization was defined as positivity to, at least, 1 aeroallergen. One hundred and twenty-eight adult patients were eligible participants for the study, and 68 cases (53.10%) were classified as having tobacco smoke exposure. Among these, most of them were secondhand smokers (50 cases, 73.50%). There was no statistically significant difference between exposure and nonexposure to tobacco smoke and indoor aeroallergen sensitization, except for the *Periplaneta americana* antigen (*p*=0.013). Most of those in the nonexposure group (34 cases, 56.70%) were classified as having intermittent allergic rhinitis, whereas the tobacco exposure group had significantly more prevalence of severe clinical symptoms. In conclusion, tobacco smoke exposure did not appear to have much influence on aeroallergen sensitization for 7 of the 8 antigens examined. However, for the *Periplaneta americana* antigen, there was a highly significant correlation with patients experiencing worsened allergic rhinitis symptoms. Overall, it was observed that allergic rhinitis patients exposed to tobacco smoke had more severe clinical symptoms. Future studies should look for other potential antigens of interest, such as mould. Implementation of public health practices reducing exposure to tobacco smoke could have benefits in allergic rhinitis patients.

## 1. Introduction

Stimulation of allergic diseases affects a variety of organ systems including respiratory, gastrointestinal, and dermatological as a result of the pathophysiology. Symptoms can affect patients in many ways including the nasal mucosa (rhinorrhea and sneezing), conjunctiva (itching and watery eyes), respiratory mucosa (cough, bronchospasm, and asthma), gastrointestinal tract (nausea, abdominal pain, and diarrhea), and skin (rashes, hives, and urticaria). Epidemiologic data show that allergic diseases have continuously increased both in developed and developing countries [1]. The prevalence of asthma and allergic rhinitis (AR) according to the recent WHO statistics comprises 334 and 400 million patients, respectively [1, 2]. The prevalence of atopic dermatitis (AD) has increased in Europe, Mideast Asia, and Africa [3]. In the US, food allergy prevalence appears to increase as well, with 1–10% of adults and 8% of children being affected [4, 5]. Different definitions of food allergy, categories of food, and inclusion and diagnostic criteria all affect different estimates of disease prevalence [6].

Direct and indirect negative impacts of AR include acute and chronic sinusitis, otitis media, sleep disruption, concentration difficulties, and behavioural disturbances [7]. There is overlap in the allergic disease spectrum, with many asthmatic patients suffering from coincident AR [8]. Affected patients experience a decreased quality of life [9, 10]. Some examples include increased school or work absence and increased health-care expenditures. In 2003, the estimated total cost related to AR in the US ranged from 2 to 5 billion US dollars [11]. Data in 2011 in the UK showed that the total cost of asthma was 1.1 billion pounds [12]. Moreover, indirect costs including loss of work day and decreased productivity are problematic for national economic and social development [13–16].

Clinical evaluation of patients through history and thorough physical examination are the main components for making the diagnosis in allergic diseases. Specific investigations are needed in confirmative diagnoses (i.e., a skin prick test or blood tests for specific IgE response to aeroallergens) [17–19]. Normally, in primary care settings, the physician does not rely on specific investigations but usually depends on the clinical information, especially for children [20]. If affected patients do undergo testing for specific aeroallergens, avoidance measures are advised. Thus, the levels of disease control could be improved with more widespread testing, and as a result, the utilization of medications for symptom control would be optimized [21]. In the general population, the skin prick testing is a benefit for defining sensitization. If a sensitized individual adapts to his/her indoor environment, the chance of clinical allergy symptoms in the future would be decreased [22–24]. A population-based case control study in Finland [25] revealed that indoor-aeroallergen sensitization in allergic asthma was 55.00% whereas it was 39.00% in the control group. In 2009, indoor-aeroallergen sensitization among Thai asthmatic children showed that 48.50% were positive to *Dermatophagoids pteronyssinus* and *Dermatophagoids farinae*, while 26.30% were positive to *Periplaneta americana*, and 7.10% were positive to cat dander [26]. In asymptomatic individuals, 42.00% were positive to, at least, one aeroallergen [27]. Most of them were sensitized to two indoor aeroallergens; house dust mite, and cockroach antigen.

As shown from many previous studies, tobacco smoke exposure increases the prevalence of allergic rhinitis [28, 29]. In addition, it also influences sensitization of both food and aeroallergens [30]. Some studies showed that tobacco smoke exposure increased sensitization [31–33]. On the other hand, a lot of researchers found an inverse impact [28, 29, 34, 35].

In daily practice, if physicians can investigate for specific information on the state of sensitization among their allergic rhinitis patients, it may help optimize their management. For example, patients may adopt avoidance measures or undergo allergen immunotherapy. Exposure to tobacco smoke may further harm these patients through sensitization to various aeroallergens. Hence, this study aims to compare indoor-aeroallergen sensitization in adults with AR between those not exposed and exposed to tobacco smoke.

## 2. Materials and Methods

### 2.1. Study Design and Sample Size

We performed a cross-sectional descriptive analytic study of indoor aeroallergen sensitization among university students and staff with AR in those exposed and not exposed to tobacco smoke. We recruited eligible participants from February 1, 2018, to March 31, 2019, at an out-patient department at Walailak University Hospital, Nakhon Si Thammarat, Thailand. By calculation, we used the formula for study the difference between 2 independent variables for numerical data as follows [36]:

(1) $$\begin{array}{c} n=\frac{{\left[\left.Z_{\alpha /2}\sqrt{2P\left(1-P\right)}+Z_{\beta }\sqrt{p_{1}\left(1-p_{1}\right)}+p_{2}\left(1-p_{2}\right)\right)\right]}^{2}}{{\left(p_{1}-p_{2}\right)}^{2}}, \end{array}$$

where *n* = sample size for each group, *p*1 = proportion part 1 (prevalence of aeroallergen sensitization in the tobacco exposure group with allergic rhinitis = 0.67 (house dust mite) [37], and *p*2 = proportion part 2 (prevalence of aeroallergen sensitization in nontobacco exposure group with allergic rhinitis = 0.37 (house dust mite) [37]. *P* = (*p*1 + *p*2) / 2 = (0.67 + 0.37) / 2 = 0.52, Z_*α*/2_*α* = 0.05 (two-tailed) = 1.96, and Z_*β*_*β* = 0.1 (one-tailed) = 1.28

The calculated sample size was 55.75 (56) per group.

Overall, we included 128 participants eligible for enrolment. This study was approved by the committee of the Institutional Review Board (IRB) of Walailak University (Approval Number: WUEC-18-016-01).

### 2.2. Eligibility Criteria

One hundred and twenty-eight participants met the inclusion criteria of adults with AR at Walailak University (staff or current students) during the study period. All of these were previously diagnosed as AR or had developed a clinical history of AR, at least, 12 months prior based on the ARIA guidelines [38] and accepted the performance of skin prick testing. The exclusion criteria included individuals who currently used antihistamines or related medications within 7 days prior to enrolment, had active skin lesions such as urticaria, eczema, and impetigo, dermatographism, uncontrolled asthma, HIV/AIDs, hepatitis B, and hepatitis C, or were unwilling to be a participant. Written informed consent was obtained from all participants before enrolment in the study.

Demographic and clinical data were collected using a questionnaire. AR severity was classified as follows: Intermittent was defined as symptoms are present for <4 days a week or for <4 consecutive weeks. Persistent was defined as symptoms are present more than 4 days a week and for more than 4 consecutive weeks. Moderate/severe persistent was defined as one or more of the following items are present with troublesome symptoms such as sleep disturbance, impairment of daily activities, leisure and/or sport, and impairment of school or work. In mild persistent, the patient had only a small amount of symptoms without troubles [39]. The status of tobacco smoke exposure was defined as a current smoker, secondhand smoker, current smoker living with an active smoker, and nonexposure. A current smoker was defined as an individual who smoked, at least, 1 cigarette/day for, at least, 1 month in the previous year before enrolment. A secondhand smoker was defined as a nonactive smoker who reported living with ≥1 smokers in the past 1 month or longer. Nonexposure was defined as never smoking in their lifetime and not living with a current smoker [35].

### 2.3. Skin Prick Test

The skin prick testing with eight common indoor aeroallergens was performed including *Dermatophagoides pteronyssinus*, *Dermatophagoides farinae*, *Periplaneta americana*, cat dander, dog dander, para grass, careless weed, and *Cladosporium spp*. All standard allergen extract panels were used (Greater Pharma, Nakhon Phathom, Thailand). Positive and negative controls including histamine and glycerinated phenol-saline, respectively, were used. The skin prick testing was performed on the volar surface of the forearm, and skin reactions were evaluated 15 minutes after the application of the skin test. Positive test results were defined as a reaction of redness and wheal as per the works Dogru et al. and Hosseini et al. [40, 41]. Individuals who had a positive test for, at least, one aeroallergen were defined as allergen sensitization and further processed for data analysis.

### 2.4. Statistical Analyses

Categorical and numerical data for the demography of all participants were described using frequency (%), mean (±SD), and range. Tobacco exposure and clinical data used frequency (%) and mean (±SD). Statistical association between the tobacco smoke exposure status and allergen sensitization and clinical severity of AR utilized the Chi-square test. All statistical analyses of the demographic and clinical characteristics data were performed using PSPP, version 1.2.0 (2016 Free Software Foundation, Inc.). The *p* value of <0.05 was considered to be statistically significant.

## 3. Results

### 3.1. Demographic Characteristics of Participants

This study was performed from February 1, 2018, to March 31, 2019, at Walailak University Hospital out-patient department. One hundred and twenty-eight AR patients were eligible participants for the study. The mean age was 22.22 ± 7.22 years and 71.10% was male. The mean BMI was 22.46 ± 4.25 kg/m^2^. One hundred and sixteen (90.60%) participants were bachelor's degree/diploma and most of all participants were university students (89.80%) (Table1).

### 3.2. Tobacco Smoke Exposure and Clinical Data

Sixty participants (46.90%) were tobacco nonexposure. The secondhand smoker, current smoker, and current smoker living with active smoker were 39.10%, 8.50%, and 5.50%, respectively. Only 48 participants (37.50%) were previously diagnosed as AR by physician. The most common comorbidity was atopic dermatitis (18.00%). Oral antihistamine was the most common medication used by participants (Table 2).

### 3.3. Sensitization to Aeroallergens in Tobacco Smoke Exposure and Nonexposure

Sensitization to eight aeroallergens in allergic rhinitis patients are shown in Table 3. Tobacco smoke exposure affected more sensitization only in cockroach (*Periplaneta americana*) aeroallergen compared to nonexposure with statistical significance (*p*=0.013). For subgroup analysis, a current smoker had more prevalence of sensitization to cat dander and *Cladosporium spp*. than a secondhand smoker with *p*=0.012 and 0.002, respectively (data not shown).

### 3.4. Prevalence of the Tobacco Smoke Status and Severity of Allergic Rhinitis

**Figure 1:**
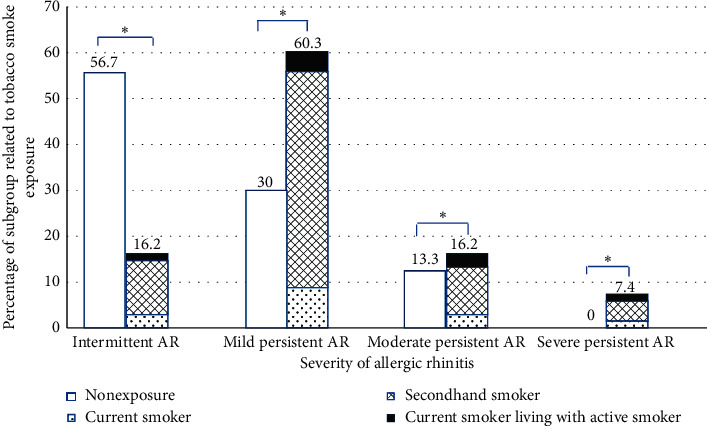
Prevalence of the tobacco smoke status and severity of allergic rhinitis, ^*∗*^*p* < 0.001.

From 60 nonexposure individuals, most of them (56.70%) were classified as intermittent allergic rhinitis. On the other hand, tobacco smoke exposure patients were distributed mostly in mild persistent allergic rhinitis with 41cases (60. 30%). The tobacco exposure group was shown to have more severe prevalence of clinical allergic rhinitis as compared to nonexposure with statistical significance (*p* < 0.001), as shown in Figure 1.

## 4. Discussion

Changes in the respiratory tract caused by exposure to tobacco smoke have been well described [42]. Tobacco smoke can directly affect the respiratory tract, leading to thickening of the lower airway walls, impaired mucociliary clearance, and altered airway immune function. This study aimed to examine further the interface of allergic symptoms in AR and tobacco smoke. Thus, we evaluated the association between tobacco smoke exposure and allergic sensitization and severity of allergic rhinitis adult patients.

After performing skin prick testing in tobacco smoke exposure and nonexposure allergic rhinitis patients, we found that only cockroach (*Periplaneta americana*) antigen from a panel of eight aeroallergens yielded higher rates of positivity, whereas the other seven aeroallergens showed no statistically significant differences. Many reports have shown that tobacco smoke increased allergen sensitization among allergic rhinitis patients [31–33]. However, some reports have shown an inverse effect of tobacco smoke on sensitization [29, 34, 35]. For example, in a US adult population cross-sectional study, a reverse relationship between tobacco smoke and inhaled allergen sensitization was found [28]. Without doubt, the issue is complex and may relate to a number of factors. One factor could be the timing of exposure to tobacco smoke as a recent systematic and meta-analysis study showed that early life exposure to environmental tobacco smoke increased the risk of sensitization [43]. The present study did not define tobacco exposure duration long enough; hence, the effect of tobacco smoke on allergens sensitization yielded mostly insignificant.

As consistent with many previous reports [29, 34], the present study demonstrated that tobacco smoke exposure led to worsened clinical severity in allergic symptoms compared to the nonexposure group. Rhinomanometry in one study examining the effect of tobacco smoke on severity of symptoms in perennial allergic rhinitis patients showed increased nasal resistance, a correlate of worsened inflammation [44]. Another study found that exposure to tobacco smoke among allergic rhinitis patients did not change severity of disease [45].

It remains to be seen whether the pathophysiologic mechanisms between smoking and IgE-mediated allergic sensitization may be distinct from those between smoking and severity of allergic rhinitis. The skin prick testing might not be affected by exposure to tobacco smoke.

The present study has some limitations including a lack of quantitative measurement for the level of tobacco exposure both in current smokers and secondhand smokers. In addition, the severity of allergic rhinitis was classified mainly by history and clinical manifestations, without objective measurement. Future research should include history of cigarette use per day, length of time that a secondhand smoker is exposed to smoking, as well as cotinine analyses in blood, urine, or even hair as a marker of exposure [46]. In addition, rhinomanometry may be useful for assessing real-time nasal conditions in the participants. Finally, testing of other aeroallergens, particularly moulds, would be worth an investigation.

## 5. Conclusions

This study demonstrated that tobacco smoke exposure worsened allergic rhinitis in patients exposed to tobacco smoke. The other aeroallergens studied appeared to have negligible impact. Complicated mechanisms of tobacco smoke exposure may affect the allergic response differently for different disorders along the allergy spectrum. We believe public health efforts concerning smoking cessation need to continue in the earnest, as this study has helped demonstrate that the detrimental health effects of tobacco smoke are far reaching.

## Figures and Tables

**Table 1:** Demographic characteristics of participants (*n* = 128).

Characteristic	Mean ± SD or *n* (%)
Age (year)	22.22 ± 7.22 (16.00–59.00)
Weight (kg)	64.28 ± 14.25 (43.40–115.00)
Height (cm)	168.87 ± 7.70 (148.00–187.00)
BMI (kg/m^2^)	22.46 ± 4.25 (16.53–35.26)
Male	91 (71.10%)
Educational level	
Junior high school	1 (0.80%)
High school	3 (2.30%)
Bachelor's degree/diploma	116 (90.60%)
Master's degree and above	8 (6.30%)
Occupation	
University student	115 (89.80%)
University staff	13 (10.20%)
Income of family (US$/month)	
≤600	31 (24.20%)
601–1,667	54 (42.20%)
1668–3,333	38 (29.70%)
≥3,334	5 (3.90%)

^*∗*^Exchange rate, Dec 15, 2019

**Table 2:** Tobacco smoke exposure and clinical data (*n* = 128).

Parameters	Mean ± SD or *n* (%)
Tobacco exposure	
Nonexposure	60 (46.90%)
Secondhand smoker	50 (39.10%)
Current smoker	11 (8.60%)
Current smoker living with active smoker	7 (5.50%)
AR diagnosis	
Previous diagnosis by physician	48 (37.50%)
First AR diagnosis	80 (62.50%)
Comorbidity	
AD	23 (18.00%)
Asthma	17 (13.30%)
Food allergy	14 (10.90%)
Allergic conjunctivitis (AC)	13 (10.20%)
Anaphylaxis	4 (3.10%)
Others^*∗*^	14 (10.90%)
Current medication	
Oral antihistamine	36 (28.10%)
Normal saline irrigation	20 (15.60%)
Intranasal corticosteroid	10 (7.80%)
Leukotriene receptor antagonist	5 (3.90%)
Intranasal decongestant	4 (3.10%)

Other ^*∗*^Insect stings and drugs allergy.

**Table 3:** Sensitization to aeroallergens in tobacco smoke exposure and nonexposure (*n* = 128).

Aeroallergens	Tobacco smoke exposure status		*p* value
	Nonexposure (*n* = 60)	Exposure (*n* = 68)	
Dp	41 (68.3%)	51 (75%)	0.403
Df	38 (63.3%)	52 (76.5%)	0.105
CR	11 (18.3%)	26 (38.2%)	0.013^*∗*^
CAT	9 (15%)	11 (16.2%)	0.855
DOG	3 (5%)	6 (8.8%)	0.398
Para grass	3 (5%)	7 (10.3%)	0.265
Careless weed	1 (1.7%)	2 (2.9%)	0.634
Clado	1 (1.7%)	2 (2.9%)	0.634

Dp, *Dermatophagoides pteronyssinus*; Df, *Dermatophagoides farinae*; CR, cockroach (*Periplaneta americana*); CAT, cat dander; DOG, dog dander; Clado, *Cladosporium spp*.

## Data Availability

The data used to support the findings of this study are included within the article.
